# Supplementary material for: Characterization of Two NMN Deamidase Mutants as Possible Probes for an NMN Biosensor
Source: Int J Mol Sci. 2021 Jun 13;22(12):6334. doi: 10.3390/ijms22126334 (PMC8231969; doi:10.3390/ijms22126334)
Supplement: Supplementary file 1 [file ijms-22-06334-s001.zip › ijms-1188376-supplementary.pdf]

Article

Characterization of two NMN deamidase mutants as possible probes for an NMN biosensor

Alessandra Camarca[1], Gabriele Minazzato[2], Angela Pennacchio[1], Alessandro Capo[1], Adolfo Amici[3], SabatoD'Auria[1,4*] and Nadia Raffaelli[2*]

1 Institute of Food Sciences, National Research Council, Via Roma 64, 83100 Avellino, Italy
2 Department of Agricultural, Food and Environmental Sciences, Polytechnic University of Marche, Via Brecce Bianche, 60131 Ancona, Italy
3 Department of Clinical Sciences DISCO, Section of Biochemistry, Polytechnic University of Marche, Via Brecce Bianche, 60131 Ancona, Italy
4 Department of Biology, Agriculture and Food Science, CNR, Piazzale Aldo Moro, 7, 00125 Rome, Italy
* Correspondence to: N. Raffaelli, Department of Agricultural, Food and Environmental Sciences, Via Brecce Bianche, 60131 Ancona, Italy, Fax: +39 71 2204 677, Tel: +39 71 2204 682, E-mail: n.raffaelli@staff.univpm.it;
S. D'Auria, Department of Biology, Agriculture and Food Science, CNR, Piazzale Aldo Moro, 7, 00125 Rome, Italy, Tel. +39-3683422770, sabato.dauria@cnr.it

Supplementary Table 1. S29A PncC fluorescence quenching (%) *vs* buffer

	Nucleotides concentration [μM]									
	**0.185**	**0.375**	**0.75**	**1.5**	**3**	**6**	**12**	**24**	**48**	**96**
**NMN**	0.48	0.58	2.58	10,.1	17.42	17.55	27.06	37.52	46.79	52.82
**NaNM**	1.79	2.56	6.47	20.83	30.74	30.87	40.52	47.93	52.28	55.20
**Na**	0	0	0	0	0	0	0	0.16	0.62	0.24
**Nam**	0.08	0.18	0.48	1.49	2.51	2.65	3.29	3.29	3.71	5.01
**NR**	0	0.12	0.80	0.96	1.84	1.98	2.29	3.13	3.51	4.48
**NAD**	0.52	0.32	0.80	0	0.19	0.32	1.35	2.54	4.58	7.97
**NADP**	0	0	0.67	1.60	2.74	2.87	3.89	4.31	6.36	9.50
**NaAD**	0	0	0	0	0	0	0	0	1.97	6.69

Supplementary Table 2. K61Q PncC fluorescence quenching (%) *vs* buffer

	Nucleotides concentration [μM]									
	**0.185**	**0.375**	**0.75**	**1.5**	**3**	**6**	**12**	**24**	**48**	**96**
**NMN**	1.09	1.84	3.19	4.87	6.26	8.11	12.12	20.07	38.03	53.35
**NaNM**	0.71	1.11	2.05	2.12	2.82	2.80	4.14	4.9	7.14	10.68
**Na**	0.49	1.2	2.11	2.66	2.89	2.81	3.26	2.74	3.69	4.46
**Nam**	0	0.34	0.87	0.68	0.91	0.26	1.15	1.04	1.14	1.57
**NR**	2.13	2.97	3.37	3.71	3.13	2.36	2.7	2.1	1.79	1.49
**NAD**	0.63	1.39	1.57	0.80	0.85	0	0.65	0.36	0.72	1.58
**NADP**	0	0.06	0.29	0.55	0.42	0	1.20	1.59	2.90	5.10
**NaAD**	0.65	1.02	1.68	2.19	2.25	2.39	3.72	4.13	5.77	8.28

Supplementary table 1 and table 2: S29A PncC and K61Q PncC fluorescence quenching (%) at increasing concentration of nucleotides. The percentages of reduction of fluorescence intensities compared to buffer, were calculated as follow: (normalized fluorescence in presence of analyte at a given [μM]/normalized fluorescence in presence of corresponding volume of buffer) *100.

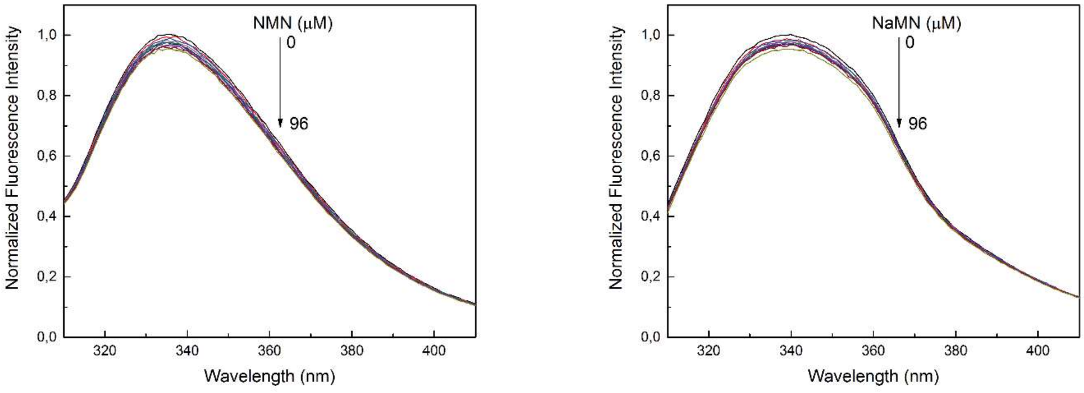

(a) (b)

GlnBP fluorescence reduction (%) vs buffer

	Nucleotide concentration [µM]								
	**0,375**	**0,75**	**1,5**	**3**	**6**	**12**	**24**	**48**	**96**
**NMN**	1,21	1,34	0,689	0,882	2,354	2,657	4,212	3,842	4,362
**NaNM**	1,463	0,699	0,375	0,567	2,273	1,958	3,227	3,267	4,358

(c)

Figure S1: Effects of NMN and NaMN on steady-state fluorescence emission of GlnBP. GlnPB (3µM) was incubated with increasing concentration of NMN, NaMN or buffer (not shown), as described for the PncC proteins. **(a)** and **(b)** steady-state fluorescence emission representative spectra **(c)** Percentages of reduction of fluorescence intensities compared to buffer, were calculated as follow: (normalized fluorescence in presence of analyte at a given [µM]/normalized fluorescence in presence of corresponding volume of buffer) *100.
